# SOLVE: A structured orthogonal latent variable framework for disentangling confounding in matrix data

**DOI:** 10.1093/biomethods/bpaf094

**Published:** 2026-01-28

**Authors:** Jialai She, Gil Alterovitz

**Affiliations:** Phillips Academy, Andover; PRIMES, Massachusetts Institute of Technology, Cambridge, Massachusetts, United States; Harvard Medical School, Harvard University, Boston, Massachusetts, United States

**Keywords:** latent factor models, computational biology, identifiability constraints, low-rank matrix factorization, confounding adjustment, matrix outcomes

## Abstract

Latent factor models are valuable in bioinformatics for accounting for unmeasured variation alongside observed covariates. Yet many methods struggle to separate known effects from latent structure and to handle losses beyond standard regression. We present a unified framework that augments row and column predictors with a low-rank latent component, jointly modeling measured effects and residual variation. To remove ambiguity in estimating observed and latent effects, we impose a carefully designed set of orthogonality constraints on the coefficient and latent factor matrices, relative to the spans of the predictor matrices. These constraints ensure identifiability, yield a decomposition in which the latent term captures only variation unexplained by the covariates, and improve interpretability. An efficient algorithm handles general non-quadratic losses via surrogates with monotone descent. Each iteration updates the latent term by truncated singular value decomposition of a doubly projected residual and refines coefficients by projections. The number of latent factors is selected by applying an elbow rule to a degrees-of-freedom-adjusted information criterion. A parametric bootstrap provides valid inference on feature-outcome associations under the regularized low-rank structure. Applied to real pharmacogenomic data, the method recovers biologically coherent gene-drug associations missed by standard factor models, such as the EGFR-inhibitor link, highlights novel candidates with plausible mechanisms, and reveals gene programs aligned with compound modes of action, including a latent unfolded-protein-response module affecting drug sensitivity. These results support the framework’s utility for precision oncology, yielding stronger biomarkers for patient stratification and deeper insight into drug resistance mechanisms.

## Graphical abstract

**Figure:**
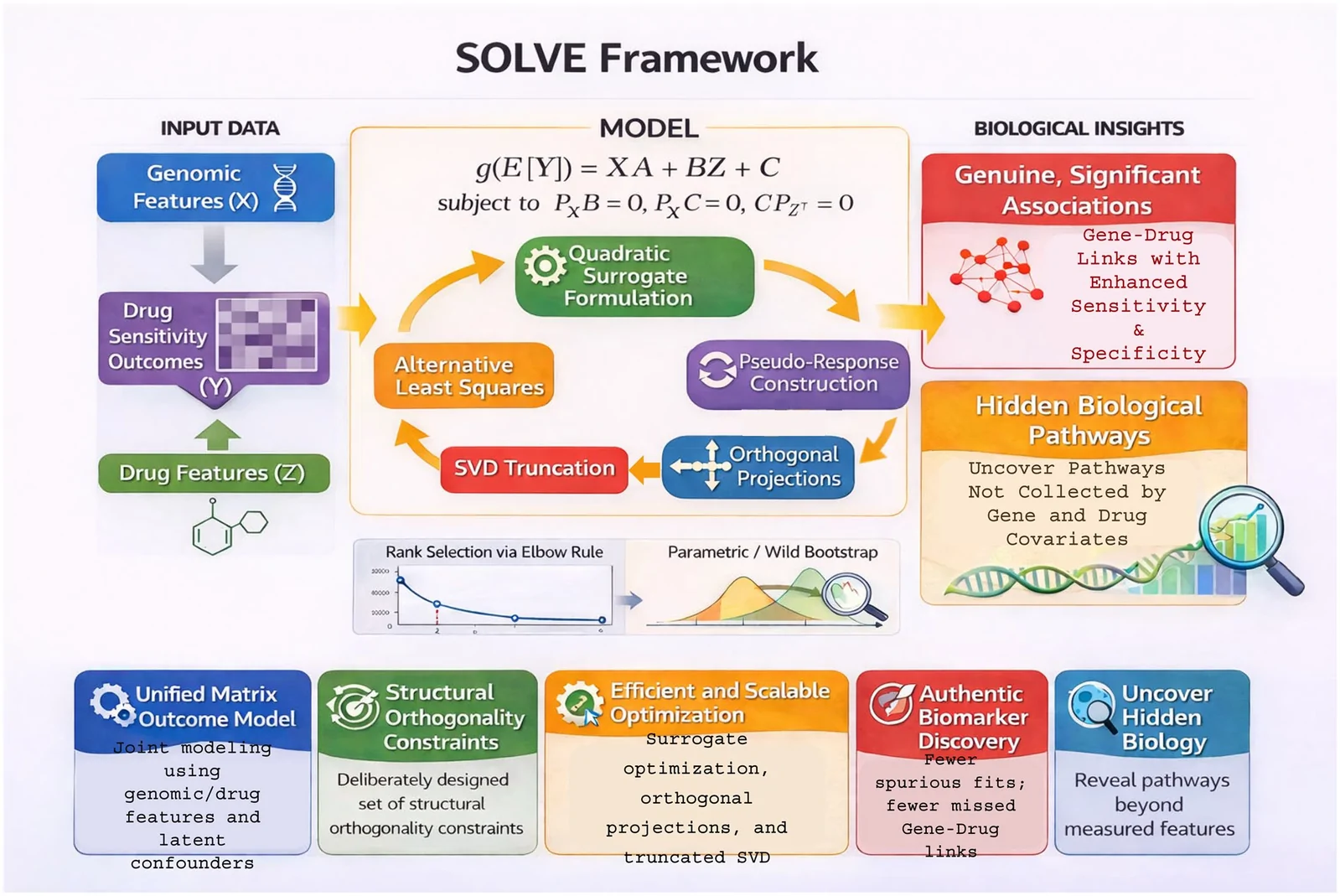

## Introduction

In many scientific fields, a central goal is to infer the effect of a predictor of interest, $x_{j}$, from a primary predictor matrix $X\in R^{n\times p}$, on a particular outcome, $y_{k}$, from an outcome matrix $Y\in R^{n\times m}$, often leveraging auxiliary column-specific data, *Z*. However, such analyses are frequently compromised by unobserved confounders—latent variables that distort estimation and inference and induce spurious associations [1, 2].

Our primary motivating example comes from *Precision Oncology*, a modern approach to cancer treatment that tailors therapies to the unique molecular profile of a patient’s tumor. Implementing this paradigm relies on preclinical discovery tools, most notably large-scale pharmacogenomic screens, such as the Cancer Cell Line Encyclopedia (CCLE) [3]. These screens systematically test drugs on hundreds of cancer cell lines—each representing a different tumor type—to discover predictive biomarkers: genomic features that predict a drug’s effectiveness.

The data from these studies yield three key matrices. First, an outcome matrix *Y*, where each entry $y_{ik}$ measures the sensitivity (or effectiveness) of the *k*-th drug on the *i*-th cancer cell line. This outcome can be a continuous measure, such as the half-maximal inhibitory concentration (IC50) or the area under the dose-response curve (AUC)—for both metrics, lower values indicate higher sensitivity. The outcome can also be converted to a binary indicator (sensitive vs. resistant) for classification purposes [4]. Second, a matrix of row-specific primary predictors *X*, which contains the rich genomic features for each cell line, such as gene expression levels or the status of specific mutations. Finally, a matrix of column-specific auxiliary predictors *Z* encodes key drug properties, such as chemical structure or molecular targets.

However, a model based solely on observed features is insufficient because drug response is governed by a multitude of *unobserved* variables reflecting latent properties of both cell lines and drugs. For instance, a cell line’s response is often determined by the activation state of critical signaling pathways (e.g., MAPK, PI3K/AKT) or the influence of the tumor microenvironment—factors rarely measured directly in high-throughput screens [5, 6]. The presence of these influential but unobserved variables creates systematic confounding that can mask true drug-gene associations or create false ones.

### Related work

Existing methods for handling multivariate confounding often fall short when the primary goal is accurate inference on specific predictor-outcome relationships (*X* to *Y*). Standard regression models simply ignore latent variables, while popular adjustment strategies typically follow a multi-step, *ad hoc* process—often employing principal component analysis (PCA) to extract latent factors from outcome data after accounting for known predictors [7]. For example, SVA [8] addresses hidden sources of variation by regressing out known predictors, decomposing the residuals, selecting informative gene subsets, and constructing surrogate variables for downstream analysis. PEER [9] uses Bayesian factor analysis with automatic relevance determination to infer the number of latent factors directly from the data. CATE [10] adjusts for confounding either through user-specified negative controls or, under a sparsity assumption, by robust regression on the bulk of the data.

However, these prevailing approaches have key drawbacks.

1. Tailored for continuous outcomes under squared-error loss, these procedures and implementations do not naturally extend to other data types, such as binary outcomes in classification, nor do they accommodate both row- *and* column-wise predictors.
2. Neither SVA nor PEER strictly separates estimated latent factors from observed predictors, which can result in **unidentifiable** parameters and complicate interpretation.
3. All these methods share a weakness rooted in their *sequential* design. By estimating the latent structure in a separate, subsequent step (rather than through joint optimization) they often fail to achieve global optimality. Indeed, as recognized in related fields such as panel data econometrics, these sequential procedures can lead to biased estimates of the coefficients of interest [11].

### A unified SOLVE framework

The latent modeling of matrix outcomes presents significant challenges, but a key insight that makes this problem tractable is the assumption that the residual variation in *Y* (not explained by the observed predictors) has a **low-dimensional** structure, a premise rooted in the biological reality that cellular response is often orchestrated by a small number of master regulators [12]. This principle of hierarchical control implies that the collective effect of thousands of unmeasured molecular events can be parsimoniously captured by a small set of latent factors. For example, a cell line may have a latent dependency on a specific survival pathway, such as Wnt signaling, a phenomenon known as non-oncogene addiction [13], while a drug may exert potent but uncharacterized off-target effects against that same pathway [14]. The resulting strong sensitivity, observed only when this specific cellular vulnerability meets the drug’s latent mechanism, creates a coherent pattern of response across the dataset. The modularity of disease networks implies that just a handful of core pathways become dominant drivers of cell survival through dysregulation [15]. Aggregating such dominant vulnerability-mechanism pairings naturally forms a low-rank structure.

The proposed framework, **S**tructured **O**rthogonal **L**atent **V**ariable **E**stimation (**SOLVE**), overcomes aforementioned challenges by jointly and explicitly modeling the contributions of unobserved latent factors alongside the observed row- and column-specific predictors within a single, unified model.

- First, a novel latent factor framework is introduced that integrates row predictors *X*, column predictors *Z*, and a low-rank latent component within a unified model. This formulation extends latent factor modeling beyond standard regression to accommodate a broad class of generalized linear models.
- To resolve model ambiguity, we impose carefully designed orthogonality constraints on the coefficient and latent factor matrices relative to the spans of the predictor matrices. These constraints ensure parameter identifiability and yield a unique decomposition where the latent structure captures only variation unexplained by known covariates, improving interpretability
- Computationally, an efficient optimization algorithm is proposed for general non-quadratic losses by constructing quadratic surrogates with guaranteed monotone descent and convergence. The method features closed-form updates: the latent factor is updated via truncated singular-value decomposition (SVD) of a doubly projected residual, while coefficient blocks are refined through simple projections, ensuring scalability to large datasets.
- Furthermore, for model selection and inference, the framework uses a data-driven elbow method on a penalized information criterion to select the optimal rank, and introduces a parametric bootstrap to compute valid *P*-values for the gene-drug coefficients—a task complicated by the regularized low-rank structure.
- Finally, applied to CCLE and GCSI, SOLVE recovers biologically coherent gene-drug associations that standard latent-factor models miss (e.g., EGFR expression linked to EGFR inhibitors) and identifies additional candidates whose activation signatures align with compound-specific modes of action. Interpreting the learned latent structure reveals an unfolded protein response (UPR) module driving drug sensitivity in GCSI—a known resistance pathway. Overall, SOLVE improves sensitivity and specificity and exposes latent biology beyond what measured covariates can explain.

## Materials and methods

### Datasets

#### Validation using simulated datasets

Synthetic datasets were constructed to evaluate a latent factor method’s performance under controlled conditions that mimic the structure of real pharmacogenomic data.

The data generation procedure is as follows. The row-specific predictors $X\in R^{n\times p}$ and column-specific predictors $Z\in R^{q\times m}$ were drawn independently from a multivariate normal distribution $N(0,\Sigma_{X})$, and the columns of *Z* were sampled from $N(0,\Sigma_{Z})$. Both $\Sigma_{X}$ and $\Sigma_{Z}$ specified as Toeplitz matrices with entries $\Sigma_{i,j}=\theta^{\left|i-j\right|}$, where we set the correlation parameter θ=0.2. The true underlying kernel coefficient matrices $A_{0}^{*}\in R^{p\times m}$ and $B_{0}^{*}\in R^{n\times q}$ (cf. (6)) were created with entries drawn from a standard normal distribution. The dimensions were set to be n=200, m=150, p=10, and q=8. The latent structure kernel $C_{0}^{*}$ was formed as the product of two standard normal matrices to have a true rank of $r^{*}=4$. The final coefficient matrices $A^{*},B^{*}$ (associated with X,Z, respectively) and latent factor matrix $C^{*}$ were obtained using (6), and correspondingly, the systematic component $\eta^{*}=XA^{*}+B^{*}Z+C^{*}$ (cf. (2)).

The outcome matrix *Y* was produced under two scenarios: (i) *Regression*. The response was given by $Y=\eta^{*}+E$, where the entries of the noise matrix *E* were drawn i.i.d. from $N(0,\sigma^{2})$. We tested three noise levels: $\sigma^{2}\in \{9,25,64\}$. (ii) *Classification*. The response matrix *Y* was binary with each element $y_{i,k}$ following a Bernoulli law with parameter $p_{i,k}=1/(1+\text{exp}(-\eta_{i,k}^{*}))$. For each setting, 50 independent datasets were generated in order to better average out noise and provide a robust evaluation.

#### Pharmacogenomic data: CCLE

The Cancer Cell Line Encyclopedia (CCLE) served as the first pharmacogenomic dataset in our study, a public resource cataloging both genomic profiles and drug responses for 148 human cancer cell lines [16]. Drug sensitivity was assessed on the basis of the area under the dose-response curve (AUC) for 24 compounds, with lower values indicating greater sensitivity. Baseline gene-expression profiles were obtained for all cell lines. To identify genes associated with drug response, we regressed the 24-drug response profile on individual gene expression, assessed significance using Wilks’ Lambda, and applied Bonferroni correction for multiple testing. This yielded 70 genes strongly associated with drug response, based on an adjusted *P*-value threshold of .05. For each drug, chemical and physicochemical descriptors were obtained through Dragon 7 software; a similar screening procedure yielded six key descriptors. Missing entries in the drug response matrix were imputed using the missForest algorithm, which leverages both gene expression and molecular descriptors as predictors [17].

#### Pharmacogenomic data: GCSI

The second pharmacogenomic dataset used in this study was from the Genentech Cell Line Screening Initiative (GCSI) [18]. A processed version available through the same MoDaC portal comprises 130 cancer cell lines, each tested against 16 drugs. Following a feature screening process similar to that for the CCLE data, 83 genes were retained for analysis; no significant molecular descriptors for the compounds were identified, resulting in a null drug-feature matrix. The task was formulated as a classification problem, for which drug responses measured as AUC were binarized into effective (AUC ≤ 0.7) or ineffective (AUC > 0.7); the threshold was selected based on [4] as a meaningful cutoff to identify cell lines responsive to treatment.

### Model, regularization, and identifiability

Let $Y\in R^{n\times m}$ be the response matrix of interest. For the (i,k)th outcome of $y_{i,k}$, a standard modeling approach in our setting is to use a linear model that incorporates row-specific predictors (e.g., sample features) and column-specific predictors (e.g., item features). Let $x_{i}\in R^{p}$ be the predictor vector for the *i*-th row, and $z_{k}\in R^{q}$ be the predictor vector for the *k*-th column with n>p,m>q.

To simplify the discussion, we begin with a generalized linear model (GLM) [19]:


(1) $$g(E[y_{i,k}])=x_{i}^{⊤}\alpha_{k}+\beta_{i}^{⊤}z_{k}$$

where *g* is a link function, and $\alpha_{k}\in R^{p}$ and $\beta_{i}\in R^{q}$ are the corresponding coefficient vectors. For binary outcomes $y_{i,k}\in \{0,1\}$, the *logistic* link g(t)=log(t/(1−t)) is commonly used, while for a regression model *g* is the identity function g(t)=t. In matrix form, this becomes the baseline model


g(E[Y])=XA+BZ,

where $X={\left[x_{1},\ldots ,x_{n}\right]}^{⊤}$, $A=[\alpha_{1},\ldots ,\alpha_{m}]$, $B={\left[\beta_{1},\ldots ,\beta_{n}\right]}^{⊤}$, and $Z=[z_{1},\ldots ,z_{m}]$.

A critical limitation of the baseline model is its failure to account for latent variables—unobserved factors that systematically influence the response. For example, in pharmacogenomics, a cell line may have a latent dependency on a specific survival pathway, while a drug may possess an uncharacterized potency against that same pathway. Similarly, in microbial genomics, it is necessary to account for factors like a species’ intrinsic metabolic fitness, as some species are inherently faster growers, and the general nutrient limitation of an environment. These unobserved effects create systematic patterns in the data that, if ignored, will distort estimation and inference for the model coefficients.

To address this, we introduce an adjustment matrix $C\in R^{n\times m}$:


(2) g(E[Y])=XA+BZ+C,

where the right-hand side represents the *systematic component*, often denoted by η [19]. Matrix *C* is intended to capture the effects of latent factors—real but unmeasured entities. A latent factor might represent the activity of a specific cancer-related signaling pathway or correspond to an individual’s ancestry from a specific subpopulation.

To quantify the effects of these underlying causal mechanisms not captured by the primary predictors, we must impose a structural assumption on *C*. In the previous microbial genomics example, one could model the latent effect using an additive structure: $c_{i,k}=a_{i}-b_{k}$ with $a_{i}$ representing a species’ intrinsic fitness and $b_{k}$ the growth limitation of an environment, which creates a rank-2 matrix. Similarly, in the pharmacogenomics example, the unobserved effect could be modeled multiplicatively as $c_{i,k}=a_{i}b_{k}$, where $a_{i}$ is a cell line’s latent dependency on the pathway and $b_{k}$ is a drug’s latent potency against it. Generalizing from this, we assume this latent structure matrix *C* has low-rank at most *r* and r≪min{n,m}. This assumption formalizes the principle that after accounting for the primary predictors *X* and *Z*, only a few dominant confounding factors remain.

However, (2) introduces a new problem: model ambiguity. For instance, one can always write $XA+BZ=XA+P_{X}GP_{Z^{⊤}}-P_{X}GP_{Z^{⊤}}+BZ=X(A+X^{+}GP_{Z^{⊤}})+(B-P_{X}GZ^{+})Z=XA^{\prime }+B^{\prime }Z$ for any matrix *G*, where $M^{+}$ denotes the Moore–Penrose pseudoinverse of *M* and $P_{M}=MM^{+}=M{\left(M^{⊤}M\right)}^{+}M^{⊤}$ gives the orthogonal projector onto the column space of *M*. To resolve the issue, we enforce carefully chosen orthogonality conditions to disentangle observed and unobserved effects:


(i)col(B)⊥col(X),   (ii)col(C)⊥col(X),   (iii)row(C)⊥ row(Z),

where col(M) and row(M) represent the column space (range) and row space of *M*, respectively. In particular, (ii) and (iii) ensures that *C* captures only the variation that cannot be explained by *X* or *Z*.

Imposing these constraints leads us to the model below, which we term **S**tructured **O**rthogonal **L**atent **V**ariable **E**stimation (**SOLVE**):


(3) $$\begin{array}{cc} & g(E[Y])=XA+BZ+C\\ \;\text{subject}\;\text{to}\; & P_{X}B=0,P_{X}C=0,CP_{Z^{⊤}}=0 \end{array}$$

where Y,X,Z are given, A,B,C are unknown and *C* is of low rank. The term *XA* represents the component of *Y* that is best explained by the primary predictors *X*. The second term captures the variation explained by the auxiliary column predictors *Z* within the space orthogonal to *X*. Finally, the third term models the low-rank latent structure in the residual variation orthogonal to both *X* and *Z*. Not only does this orthogonal structure ensure that the unique contributions of the observed predictors and latent factors are identifiable for proper inference, but it also provides computational tractability (cf. Theorem 1).

For regression, where *g* is the identity function, (3) can be expressed as


(4) $$\begin{array}{c} \;Y=XA+BZ+C+E\;\;s.t.\;P_{X}B=0,P_{X}C=0,CP_{Z^{⊤}}=0 \end{array}$$

For binary outcomes $y_{i,k}\in \{0,1\}$, the *logistic* link gives


(5) $$\text{log}\;\left(\frac{E[y_{i,k}]}{1-E[y_{i,k}]}\right)=x_{i}^{⊤}\alpha_{k}+\beta_{i}^{⊤}z_{k}+c_{i,k}$$

subject to the same orthogonality constraints. This logistic model is relevant for classification problems common in bioinformatics.

### Estimation, tuning, and inference

To simplify the problem, we introduce a *reparametrization* motivated by linear space constraints:


(6) $$\{\begin{array}{c} A=A_{0}\\ B=P_{X}^{⊥}B_{0}\\ C=P_{X}^{⊥}C_{0}P_{Z^{⊤}}^{⊥}, \end{array}$$

which yields the optimization problem:


(7) $$\begin{array}{c} \underset{\left(A_{0},B_{0},C_{0}\right)}{\min}l(\eta ;Y,X,Z)\\ \;s.t.\;\eta =XA_{0}+P_{X}^{⊥}B_{0}Z+P_{X}^{⊥}C_{0}P_{Z^{⊤}}^{⊥},\text{rank}(C_{0})\leq r. \end{array}$$

Here, l(η;Y,X,Z) or l(η) for short, is a general loss function to quantify the error between the model output η and the observed outcome *Y* (given X,Z).

In this section, we derive an optimization-based iterative algorithm that is both easy to implement and guaranteed to converge, *without* restricting *l* to a negative log-likelihood from a GLM.

### Closed-form solution for regression

For the standard regression case with a squared Frobenius norm loss $l(\eta )=∥Y-\eta {∥}_{F}^{2}/2$, it is tempting to solve the problem via an *alternating* procedure: repeatedly performing multivariate regression to update *A* and *B* and applying PCA to estimate *C* until convergence. Perhaps surprisingly, however, SOLVE admits a joint globally optimal *closed-form* solution for all parameters.

Let the singular value decomposition (SVD) of $A\in R^{n\times m}$ be $A=UDV^{⊤}$ where U,V are orthogonal matrices: $U^{⊤}U=I,V^{⊤}V=I$ and *D* is a diagonal matrix of the singular values in non-increasing order.

For a given rank parameter r≤min{n,m}, define a truncated SVD operator S(A;r) as


(8) $$S(A;r)=U_{r}D_{r}V_{r}^{⊤}$$

where $U_{r},V_{r}$ are the matrices formed by the first *r* columns of U,V, respectively, and $D_{r}$ is the diagonal matrix obtained from *D* by retaining only its largest *r* singular values.

Theorem 1. *The rank-restricted least squares problem*
 

(9) $$\underset{\left(A_{0},B_{0},C_{0}\right)}{\min}\frac{1}{2}∥Y-XA_{0}-P_{X}^{⊥}B_{0}Z-P_{X}^{⊥}C_{0}P_{Z^{⊤}}^{⊥}{∥}_{F}^{2}\;s.t\;.\;\text{rank}(C_{0})\leq r$$

*admits a globally optimal solution* $\left({\hat{A}}_{0},{\hat{B}}_{0},{\hat{C}}_{0}\right)$  *given explicitly by*
 

(10) $$\{\begin{array}{c} {\hat{C}}_{0}=S(P_{X}^{⊥}YP_{Z^{⊤}}^{⊥};r)\\ {\hat{A}}_{0}={\left(X^{⊤}X\right)}^{+}X^{⊤}Y\\ {\hat{B}}_{0}=YZ^{⊤}{\left(ZZ^{⊤}\right)}^{+}. \end{array}$$

The proof is presented in the Appendix. Based on (6), the solutions $\hat{A},\hat{B},\hat{C}$ can be obtained.

The existence of a non-iterative, closed-form solution to the nonconvex optimization problem (9) is a contribution with practical implications. Many advanced statistical models, particularly those involving latent variables, depend on iterative optimization algorithms such as Expectation-Maximization (EM) or Alternating Least Squares (ALS) or sampling-based methods like Markov Chain Monte Carlo (MCMC). These iterative procedures can be computationally intensive, highly sensitive to initialization parameters, and may converge to local optima rather than the global optimum. In contrast, a closed-form solution with theoretical guarantees is computationally more efficient and scalable.

### Iterative optimization via surrogates

One may encounter a non-quadratic loss in (7)—a common example is the logistic loss for binary data under the logistic link (5):


(11) $$l(\eta )=-\langle Y,\eta \rangle +\sum_{1\leq i\leq n,1\leq k\leq m}b(\eta_{i,k}),$$

where the so-called “cumulant” b(t)=log(1+exp(t)) and g(t)=log(t/(1−t), the logistic link in (5). In such GLMs, a closed-form solution rarely exists. Fortunately, we can develop an optimization algorithm based on a surrogate function and leverage a closed-form regression solution (cf. Theorem 1) to update the estimate.

We assume the loss gradient enjoys Lipschitz continuity: for any $\eta_{1},\eta_{2}\in R^{n\times m}$,


(12) $$∥\nabla l(\eta_{1})-\nabla l(\eta_{2}){∥}_{F}\leq L∥\eta_{1}-\eta_{2}{∥}_{F}$$

for some L>0, where $∥\cdot {∥}_{F}$ denotes the Frobenius norm. For instance, L=1/4 for the logistic loss since $b^{\prime \prime }(\cdot )=\text{exp}(\cdot )/{\left(1+\text{exp}(\cdot )\right)}^{2}\leq 1/4$, and L=1 for the standard squared error loss $l(\eta )=∥\eta -Y{∥}_{F}^{2}/2$.

Define a surrogate function for l(η) at $\eta^{-}$ as


(13) $$g(\eta ,\eta^{-})=l(\eta^{-})+\langle \nabla l(\eta^{-}),\eta -\eta^{-}\rangle +\frac{\rho }{2}∥\eta -\eta^{-}{∥}_{F}^{2}.$$

Starting with an arbitrary initial point $\left(A_{0}^{\left(0\right)},B_{0}^{\left(0\right)},C_{0}^{\left(0\right)}\right)$ with $\text{rank}(C_{0}^{\left(0\right)})\leq r$, we define an iterative algorithm where the (t+1)-th iterate (t≥0) is the solution to


(14) $$\begin{array}{cc} & \left(A_{0}^{\left(t+1\right)},B_{0}^{\left(t+1\right)},C_{0}^{\left(t+1\right)})=\arg\underset{\left(A_{0},B_{0},C_{0}\right)}{\min}g(\eta ,\eta^{\left(t\right)}\right)\\ & \;\;s.t.\;\eta =XA_{0}+P_{X}^{⊥}B_{0}Z+P_{X}^{⊥}C_{0}P_{Z^{⊤}}^{⊥},\text{rank}(C_{0})\leq r. \end{array}$$

The following theorem states that when ρ is chosen properly large (e.g., 1/4 in classification), the algorithm converges, with its rigorous proof presented in the Appendix.

Theorem 2. *Suppose the loss function l has an L-Lipschitz continuous gradient as in (12). Starting with any feasible initial point* $\left(A_{0}^{\left(0\right)},B_{0}^{\left(0\right)},C_{0}^{\left(0\right)},\eta^{\left(0\right)}\right)$*, let the sequence of iterates* $\left(A_{0}^{\left(t+1\right)},B_{0}^{\left(t+1\right)},C_{0}^{\left(t+1\right)},\eta^{\left(t+1\right)}\right)$  *be defined by (14) with an inverse step size* ρ≥L*. Then for any* t≥0*, we have* $\text{rank}(C_{0})\leq r$  *and*
 

(15) $$l(\eta^{\left(t\right)})-l(\eta^{\left(t+1\right)})\geq \frac{\rho -L}{2}∥\eta^{\left(t\right)}-\eta^{\left(t+1\right)}{∥}_{F}^{2}$$

*Consequently, the sequence of objective values* $\left\{l(\eta^{\left(t\right)}\right\}$  *is non-increasing and thus convergent, and under* ρ>L*, the difference between consecutive iterates converges to zero, i.e.,* $∥\eta^{\left(t\right)}-\eta^{\left(t+1\right)}{∥}_{F}\to 0$  *as* t→∞.

Now, let’s simplify (14).

$$\begin{array}{cc} g(\eta ,\eta^{-}) & =l(\eta^{-})+\langle \nabla l(\eta^{-}),\eta -\eta^{-}\rangle +\frac{\rho }{2}∥\eta -\eta^{-}{∥}_{F}^{2}\\ & =l(\eta^{-})+\langle \nabla l(\eta^{-}),-\eta^{-}\rangle +\frac{\rho }{2}∥\eta +\frac{1}{\rho }\nabla l(\eta^{-})-\eta^{-}{∥}_{F}^{2}\\ & =\frac{\rho }{2}∥\eta -(\eta^{-}-\frac{1}{\rho }\nabla l(\eta^{-})){∥}_{F}^{2}+c(\eta^{-}) \end{array}$$

where $c(\eta^{-})$ does not depend on η. The surrogate function *g* becomes simply *quadratic* in η. Introducing the pseudo-response matrix $\xi^{-}=\eta^{-}-\nabla l(\eta^{-})/\rho$, held fixed in every iteration, reduces the per-iteration optimization to solving a constrained regression.

Define


(16) $$\xi^{\left(t\right)}:=\eta^{\left(t\right)}-\frac{1}{\rho }\nabla l(\eta^{\left(t\right)}).$$

From Theorem 1, the iterate updates are given by


(17) $$\{\begin{array}{c} {\hat{C}}_{0}^{\left(t+1\right)}=S(P_{X}^{⊥}\xi^{\left(t\right)}P_{Z^{⊤}}^{⊥};r)\\ {\hat{A}}_{0}^{\left(t+1\right)}={\left(X^{⊤}X\right)}^{+}X^{⊤}\xi^{\left(t\right)}\\ {\hat{B}}_{0}^{\left(t+1\right)}=\xi^{\left(t\right)}Z^{⊤}{\left(ZZ^{⊤}\right)}^{+} \end{array}$$

for all t≥0.

An outline of our SOLVE algorithm is as follows.

Algorithm 1 The SOLVE algorithm

**Require:**  Y,X,Z, *r*, ρ, and $\left(A_{0}^{\left(0\right)},B_{0}^{\left(0\right)},C_{0}^{\left(0\right)}\right)$ with $\text{rank}(C_{0}^{\left(0\right)})\leq r$ (e.g., all zero matrices).

1: t←0, $\eta^{\left(0\right)}=XA_{0}^{\left(0\right)}+P_{X}^{⊥}B_{0}^{\left(0\right)}Z+P_{X}^{⊥}C_{0}^{\left(0\right)}P_{Z^{⊤}}^{⊥}$

2: **while** not converged **do** 

3: $\xi^{\left(t\right)}\leftarrow \eta^{\left(t\right)}-\frac{1}{\rho }\nabla l(\eta^{\left(t\right)})$

4:  $C_{0}^{\left(t+1\right)}\leftarrow S(P_{X}^{⊥}\xi^{\left(t\right)}P_{Z^{⊤}}^{⊥},r)$

5:  $A_{0}^{\left(t+1\right)}\leftarrow {\left(X^{⊤}X\right)}^{+}X^{⊤}\xi^{\left(t\right)}$

6:  $B_{0}^{\left(t+1\right)}\leftarrow \xi^{\left(t\right)}Z^{⊤}{\left(ZZ^{⊤}\right)}^{+}$

7:  $\eta^{\left(t+1\right)}\leftarrow XA_{0}^{\left(t+1\right)}+P_{X}^{⊥}B_{0}^{\left(t+1\right)}Z+P_{X}^{⊥}C_{0}^{\left(t+1\right)}P_{Z^{⊤}}^{⊥}$

8:  t←t+1

9: **end while** 

10: $A^{\left(t\right)}\leftarrow A_{0}^{\left(t\right)}$, $B^{\left(t\right)}\leftarrow P_{X}^{⊥}B_{0}^{\left(t\right)}$, $C^{\left(t\right)}\leftarrow P_{X}^{⊥}C_{0}^{\left(t\right)}P_{Z^{⊤}}$

11: **return**  $\left({\hat{A}}_{0},{\hat{B}}_{0},{\hat{C}}_{0})=(A_{0}^{\left(t\right)},B_{0}^{\left(t\right)},C_{0}^{\left(t\right)}\right)$, $\left(\hat{A},\hat{B},\hat{C})=(A^{\left(t\right)},B^{\left(t\right)},C^{\left(t\right)}\right)$.

For the GLM loss in (11), $\nabla l(\eta^{\left(t\right)})=b^{\prime }(\eta^{\left(t\right)})-Y$, where $b^{\prime }$ is applied componentwise. For standard regression, where $b^{\prime }(t)=t$ and L=1, setting ρ=1 reduces the pseudo-response to $\xi^{\left(t\right)}=Y$, allowing for convergence in a single step. For logistic regression, where $b^{\prime }(t)=1/(1+\text{exp}(-t))$ and L=1/4, any ρ≥1/4 ensures convergence. In general, the algorithm converges rapidly in practice.

### Rank selection

The sole tuning parameter in the SOLVE model is the rank *r* of the latent structure matrix *C*, which determines the model’s complexity. To select the optimal rank from the data, we propose using an information criterion that balances model fit with model complexity. Specifically, for a given rank *r*, the Akaike Information Criterion (AIC) [20] is defined as $\text{IC}(r)=-2\cdot \;\text{log}\;L(\hat{A},\hat{B},\hat{C})+2\cdot \text{DF}(r),$ where log L is the log-likelihood of the fitted model and DF(r) is the effective degrees of freedom. For the logistic regression case, the loss corresponds to the model deviance: $-2\;\text{log}\;L(\hat{A},\hat{B},\hat{C})=-2\sum y_{i,k}{\hat{\eta }}_{i,k}+2\sum \;\text{log}\;(1+\text{exp}({\hat{\eta }}_{i,k}))$ (in implementation, we use the numerically stable “softplus” function, defined as max(0,z)+log (1+exp(−|z|)), as a substitute for log (1+exp(z)) to improve computational stability). For the regression case under the assumption of Gaussian errors, $-2\;\text{log}\;L(\hat{A},\hat{B},\hat{C})=mn\;\text{log}(∥Y-\hat{\eta }{∥}_{F}^{2}/nm)$ (up to additive constants). The degrees of freedom for the SOLVE model are given by the total number of free parameters:


DF(r)=pm+nq+r(n+m−r).

Here, *pm* and *nq* are the number of parameters in *A* and *B*, respectively (which are constant when varying *r*), and r(n+m−r) is the number of free parameters in the rank-*r* matrix *C*.

**Figure 1:**
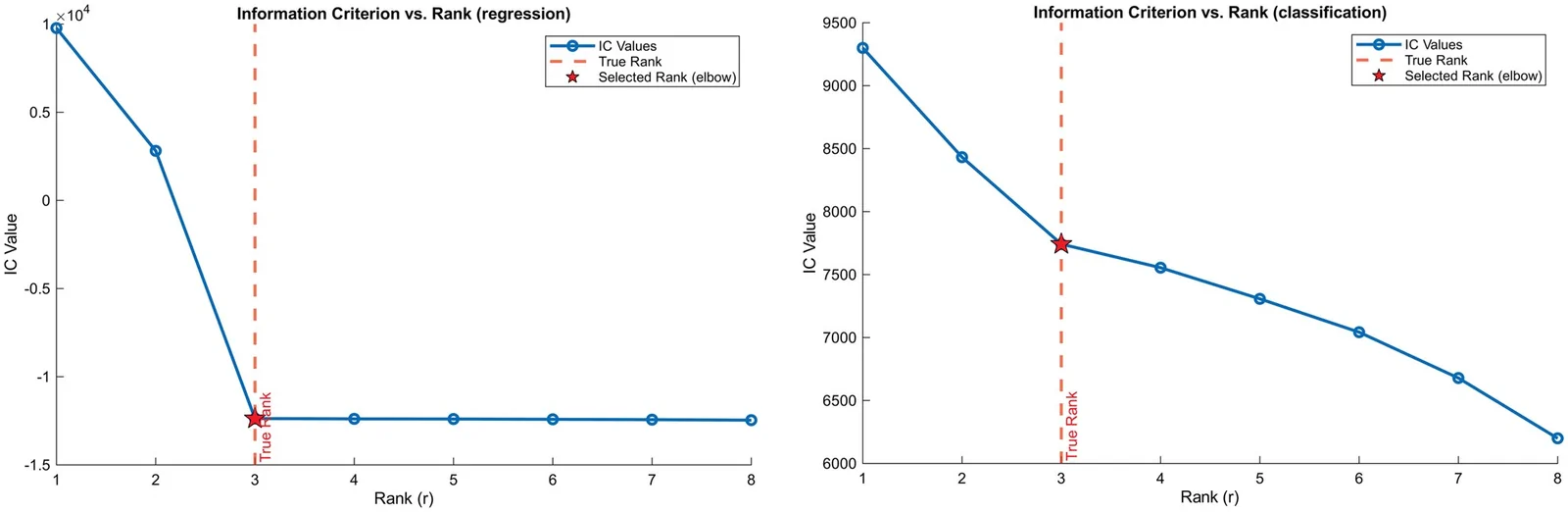
Illustration of the elbow method for selecting matrix rank in both regression (left) and logistic regression (right) models. Simulation uses n=100,m=100,p=5,q=4 and true rank r=3.

The practical procedure for rank selection is to fit the SOLVE model over a pre-specified grid Λ of candidate ranks, e.g., $\Lambda =\{1,2,\ldots ,r_{\max}\}$. Standard practice selects the rank that minimizes the information criterion, $\hat{r}=\arg\min_{r\in \Lambda }\text{IC}(r)$, but we find this approach can *overfit* by selecting a rank that is too high. Our recommendation is to use the “elbow” point: plotting the information criterion IC(r) against the rank *r* and identifying the rank at which the rate of decrease in the IC value sharply flattens (cf. Fig. 1). The underlying principle is that the singular values associated with the true signal are substantially larger than those generated by noise, creating a clear gap or elbow that marks the transition from signal to noise and reveals the effective rank. Similar ideas have also been employed in contexts such as determining the number of clusters or principal components [21]. In implementation, for an objective and reproducible selection, we first normalize both axes and then compute the perpendicular distance from each point on the curve to the straight line connecting the endpoints. The elbow point is then defined as the point with the maximum perpendicular distance from this line, corresponding to the position of greatest curvature. In our experience, this data-driven tuning robustly balances model fit and complexity, capturing low-dimensional latent structure without overfitting.

### Inference via bootstrap

Standard methods for statistical inference, which rely on deriving the asymptotic distribution of estimators, are not readily applicable to the SOLVE method. The presence of the low-rank constraint on the latent matrix *C* (which is discrete and non-differentiable) introduces challenges related to the incidental parameters problem, where the number of parameters grows with the sample size [11]. To address this, we use parametric bootstrap and wild bootstrap [22, 23] to construct valid confidence intervals.

The procedure begins with fitting the SOLVE model to the original data (Y,X,Z) using the rank selected via the information criterion as discussed earlier, yielding the estimats $\hat{A},\hat{B},\hat{C}$ and $\hat{\eta }=X\hat{A}+\hat{B}Z+\hat{C}$. Then a bootstrap response matrix $Y^{\left(b\right)}$ is constructed and the model is refit. This is repeated for a large number of bootstrap samples, b=1,…,B. For example, for the classification case with binary outcomes, we convert the fitted logits $\hat{\eta }$ to probabilities via $g^{-1}$: $\hat{P}=1/(1+\text{exp}(-\hat{\eta }))$, and then generate each element of the bootstrap data matrix by drawing from the corresponding Bernoulli distribution, $y_{i,k}^{\left(b\right)}∼\text{Bernoulli}({\hat{p}}_{i,k})$. For regression, we use a wild bootstrap to robustly handle potential heteroskedasticity: generate residuals $\hat{E}=Y-\hat{\eta }$ and apply independent Rademacher weights $W^{\left(b\right)}$. The bootstrap sample is $Y^{\left(b\right)}=\hat{\eta }+W^{\left(b\right)}{}^{\circ}\hat{E}$, where ° denotes element-wise multiplication.

After generating the bootstrap data $Y^{\left(b\right)}$ for either case, the SOLVE algorithm is refit on $\left(Y^{\left(b\right)},X,Z\right)$ to obtain bootstrap parameter estimates $\left({\hat{A}}^{\left(b\right)},{\hat{B}}^{\left(b\right)}\right)$. The resulting collection of estimates is then used to construct confidence intervals, enabling inference on the primary parameters via resampling.

## Results and discussion

### Performance comparison in simulations: accuracy and rank recovery

On each generated dataset, we applied the SOLVE algorithm (cf. Algorithm 1) and selected the optimal rank by the elbow method from a candidate grid of {1,…,10}. For comparison, we also evaluated SVA [8] and CATE [10] using their standard R package implementations. Since these methods do not include column-specific predictors, we first regressed *Y* on *Z* and then applied the algorithms to the resulting residual matrix. Both SVA and CATE were configured to automatically select the number of latent factors.

To measure estimation accuracy, we computed the relative Frobenius norm error between the true parameters and their estimates, e.g., $100\times ∥\hat{A}-A^{*}{∥}_{F}/∥A^{*}{∥}_{F}$. The performance in identifying the correct number of latent factors was measured by the percentage of experiments where the estimated rank $\hat{r}$  *exactly* matches the true rank $r^{*}$. The total computational time was calculated by summing the execution time of the model-fitting and tuning of all 50 replicates. A summary of the experimental results is presented in Table 1.

**Table 1.** Simulation results comparing data-driven SOLVE, SVA, and CATE under regression (with varying noise levels) and logistic regression.

		Method		
	Metric	SOLVE	SVA	CATE
$\sigma^{2}=9$	Error on *A*	7.71 ± 0.16	24.12 ± 0.05	30.38 ± 0.52
	Error on *B*	8.88 ± 0.16	28.70 ± 0.24	28.70 ± 0.24
	Error on η	9.28 ± 0.09	16.82 ± 0.04	9.29 ± 0.09
	Rank recovery	100%	100.0%	100.0%
	Time	3.6	28	246
$\sigma^{2}=25$	Error on *A*	12.91 ± 0.23	26.19 ± 0.11	32.21 ± 0.72
	Error on *B*	14.79 ± 0.27	31.18 ± 0.31	31.18 ± 0.31
	Error on η	15.85 ± 0.17	20.21 ± 0.13	15.87 ± 0.17
	Rank recovery	100%	92.0%	100.0%
	Time	3.6	31	282
$\sigma^{2}=64$	Error on *A*	20.68 ± 0.32	30.80 ± 0.29	30.88 ± 0.61
	Error on *B*	23.66 ± 0.50	36.47 ± 0.62	36.47 ± 0.62
	Error on η	26.56 ± 0.58	28.17 ± 0.92	25.22 ± 0.24
	Rank recovery	68.0%	8.0%	0.0%
	Time	3.6	32	275
Bernoulli	Error on *A*	34.29 ± 0.30	—	—
	Error on *B*	35.32 ± 0.37	—	—
	Error on η	36.34 ± 0.35	—	—
	Rank recovery	86.0%	—	—

Total computational time is reported in regression for method comparison. Errors are multiplied by 100 and are reported as mean ± standard deviation over 50 experiments.

In the regression setting, both SVA and CATE provide reasonable estimates for the overall signal η, particularly at lower noise levels. However, they struggle to accurately disentangle and estimate the primary coefficient matrices *A* and *B*. For example, at the noise level $\sigma^{2}=9$, the estimation errors for *A* are high for both SVA (24.12) and CATE (30.38). In contrast, SOLVE offers a substantial improvement, achieving an error of only 7.71 for *A*—a more than three-fold improvement. This advantage in recovering the primary coefficients persists as the noise level increases. At $\sigma^{2}=64$, the ability of SVA and CATE to recover the correct rank collapses. SOLVE, however, proves far more robust, maintaining a decent rank recovery rate and providing significantly more accurate estimates for *A* and *B*. In the more difficult classification setting, to which SVA and CATE are not applicable, SOLVE continues to perform well. The estimation errors are higher (as anticipated due to the information loss from binary outcomes) but remain controlled. The rank selection procedure remains effective, correctly identifying the true rank in 86% of simulations.

SOLVE also delivers a substantial computational advantage, as shown in Table 1. To examine scalability more directly, Table 2 reports average runtimes for model fitting and tuning under varying (n,m) while holding other settings fixed (e.g., σ=3.0, p=10, q=8). The results are striking: SOLVE runs about 5× faster than SVA and nearly 50× faster than CATE across all tested dimensions. This persistent gap highlights SOLVE’s strong scalability and its practical value for larger problems.

**Table 2.** Average computational time (in seconds) for SOLVE, SVA, and CATE across varying data dimensions (n,m).

	Mean Runtime (seconds)		
	SOLVE	SVA	CATE
n=200, m=150	0.090	0.469	4.220
n=200, m=300	0.184	1.316	11.477
n=400, m=150	0.143	1.774	11.648
n=400, m=300	0.572	3.703	25.455

Results are averaged over 50 experiments.

Overall, the simulation results demonstrate that the proposed SOLVE algorithm more efficiently and accurately recovers the true coefficient matrices and latent structure from confounded matrix data, even in high-noise and non-Gaussian settings.

### Uncovering biologically coherent gene-drug associations

**Figure 2:**
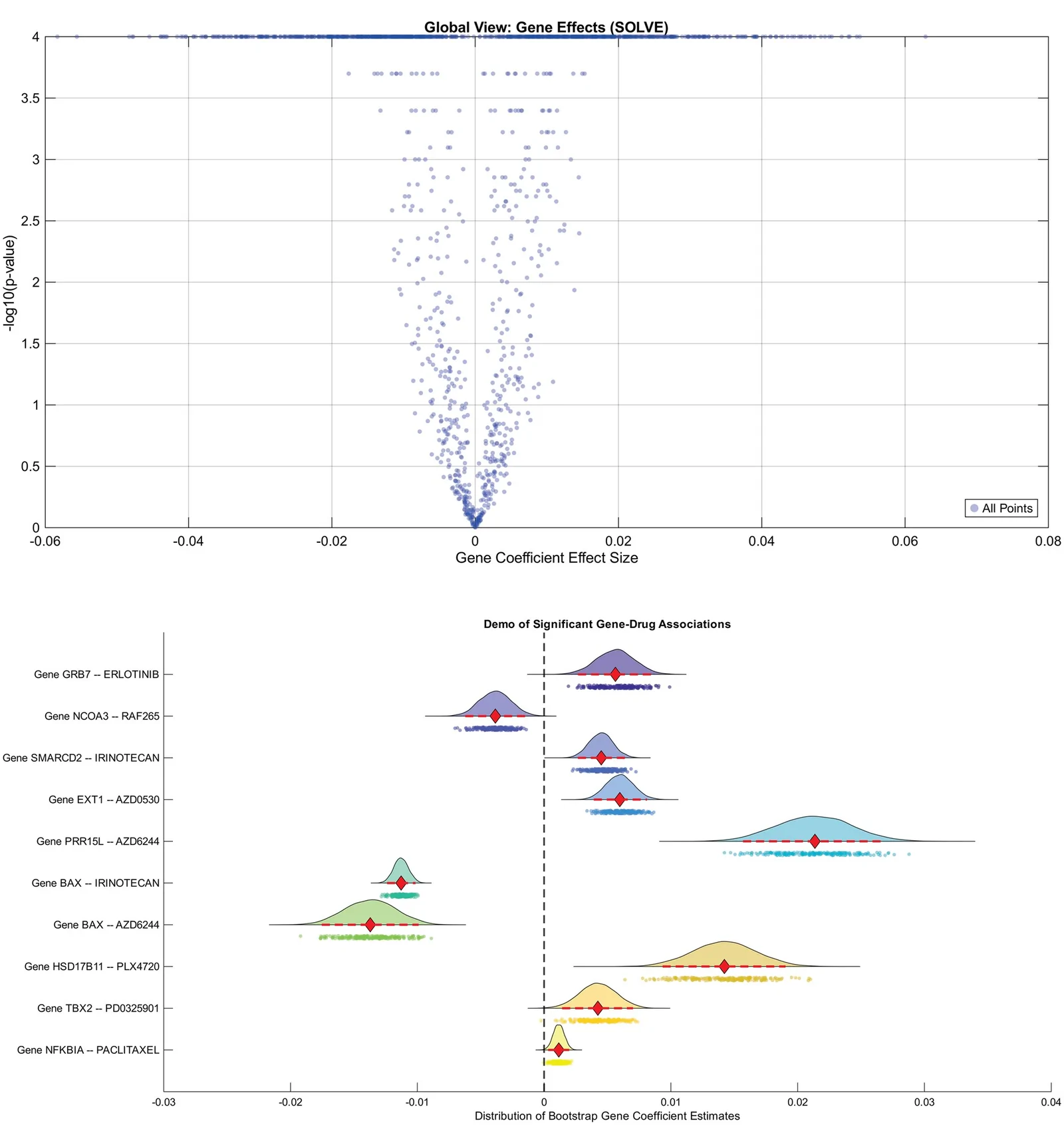
CCLE data. The top panel provides a global overview of the effect size and significance of all estimated gene-drug coefficients ($\hat{A}$) by SOLVE. The bottom panel shows bootstrap distributions for 10 top discoveries by SOLVE that were missed by CATE (CATE *P*-values > .98, SOLVE *P*-values < 5e-3), where row labels denote specific gene-drug pairs.

Using the CCLE data, we fit SOLVE to standardized predictor matrices (intercepts included). We identified 12 latent factors via rank tuning and performed inference using 1000 bootstrap samples (cf. Materials and Method). The volcano plot in Fig. 2 summarizes the resulting effect sizes and *P*-values. A comparative analysis against the CATE package [10] revealed numerous significant gene-drug interactions that were uniquely discovered by SOLVE, some illustrated in the figure.

We demonstrate two primary strengths of the SOLVE model. The first is its ability to produce biologically coherent results by explicitly modeling the shared properties (*Z*) of related drugs, unlike methods that treat each compound independently. This advantage is evident in its analysis of two therapeutic compounds, the MEK inhibitors, PD-0325901 and AZD6244. Leveraging their strongly correlated molecular descriptors in the drug-feature matrix (correlation >0.9), SOLVE translates this structural similarity into nearly identical gene-interaction profiles (Pearson r=0.988). In contrast, the CATE-derived gene profiles for these two drugs were only moderately correlated (r=0.634). SOLVE uses shared molecular features of drugs to predict gene associations that are more pharmacologically accurate.

Another key advantage of the SOLVE model is its increased statistical power, driven in part by effective characterization of latent factors (*C*), which enables detection of biologically meaningful gene-drug associations often missed by CATE. For example, SOLVE identified a highly significant association between GRB7 expression and sensitivity to the drug Erlotinib (*P* = 4e-4 for SOLVE vs. .98 for CATE). Erlotinib is a well-established EGFR inhibitor, and GRB7 encodes an adaptor protein mediating signaling via EGFR and HER2 pathways; its overexpression is a marker of pathway hyperactivation linked to cancer progression and resistance [24]. Similarly, SOLVE revealed a strong association between SMARCD2 expression and Irinotecan sensitivity (*P* = 1e-4 for SOLVE vs. .99 for CATE). Irinotecan is a topoisomerase I inhibitor that induces DNA damage, and SMARCD2 encodes a core SWI/SNF complex subunit essential for the DNA damage response [25]. Notably, loss of SWI/SNF function has been shown to sensitize cancer cells to topoisomerase inhibitors [26].

Interestingly, beyond recovering established drug-target interactions, SOLVE uncovers new, biologically informative associations. For Erlotinib, SOLVE identified both the canonical target EGFR and novel candidates genes such as PRR15L as highly significant predictors of drug sensitivity (both *P* = 1e-4), whereas CATE failed to detect the latter (*P* = .30). High PRR15L expression may signal a cell’s functional reliance on EGFR-driven survival signaling [27, 28]. This highlights an important insight: biomarkers reflecting downstream pathway dependence can complement target expression in predicting therapeutic response. Overall, compared with conventional approaches, SOLVE’s ability to borrow information across mechanistically related drugs and effectively reduce structured noise by modeling latent factors leads to greater sensitivity for discovering such nuanced gene-drug relationships.

### Improved power, specificity, and latent pathway discovery in GCSI

**Figure 3:**
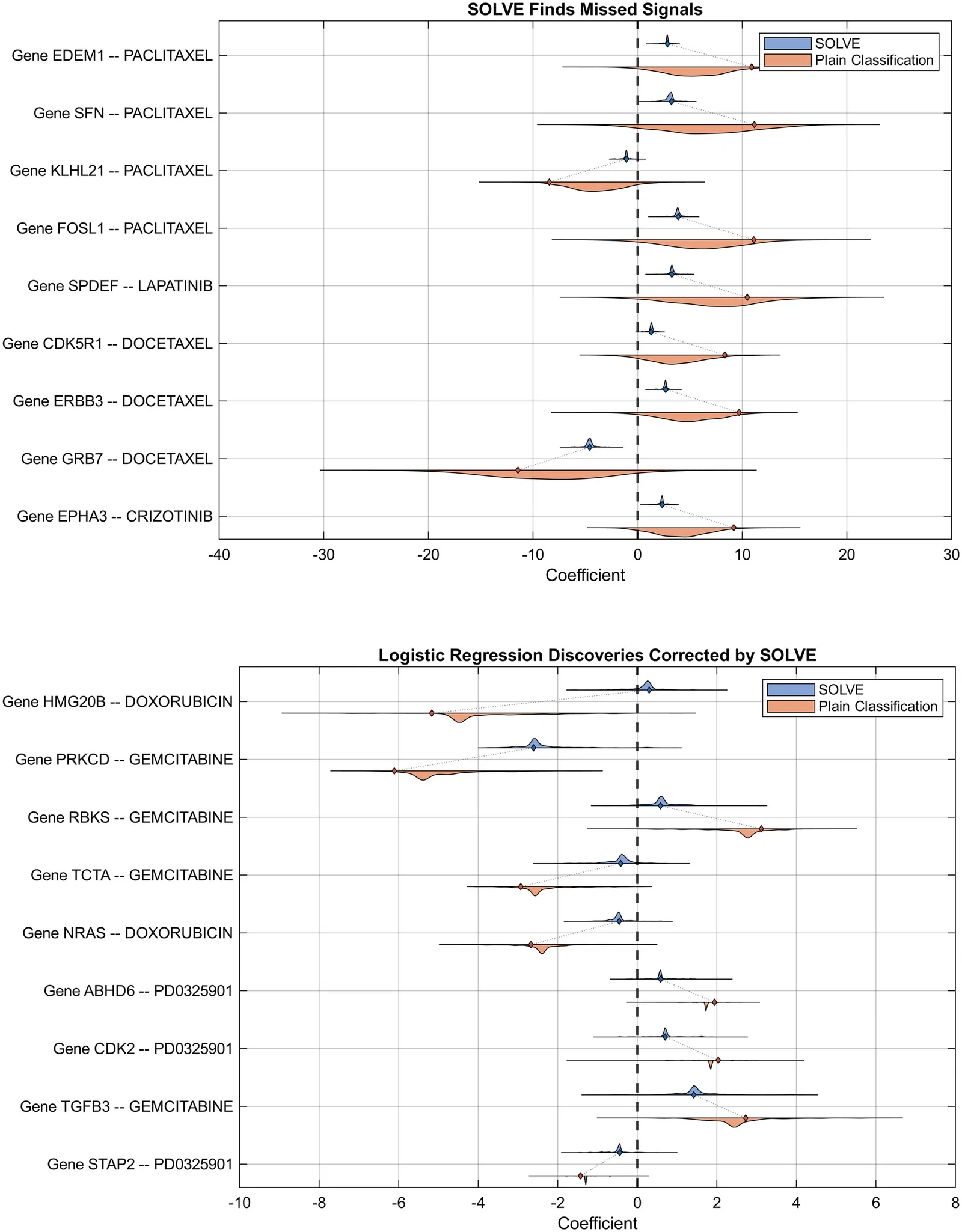
GCSI data. Comparison of SOLVE and standard logistic regression (without latent factors) in identifying significant gene-drug pairs (formatted as Gene Name—Drug Name). Top: Raincloud plots of the top significant gene-drug associations identified by SOLVE but missed by standard logistic regression. Bottom: Examples where logistic regression findings were corrected by SOLVE to lower significance.

For the classification task on the GCSI dataset, we compared SOLVE with logistic regression, using 1000 bootstrap samples to assess the significance of gene-drug associations. (Other prominent latent factor methods like SVA and CATE were excluded, as they are designed for regression problems.) Figure 3 shows a demonstration.

The top panel of Fig. 3 highlights SOLVE’s increased statistical power to detect biologically meaningful associations missed by standard methods. A prime example is the link between SFN (Stratifin, also known as 14-3-3σ) expression and the drug Paclitaxel, which SOLVE identified as highly significant (*P* = 1e-3), whereas standard logistic regression failed to distinguish this signal from noise (*P* > .14).

Paclitaxel induces mitotic arrest through microtubule stabilization [29]. SFN is a p53-regulated checkpoint factor that prevents mitotic entry by sequestering the CDC2-Cyclin B1 complex in the cytoplasm under stress [30]. Because elevated SFN enforces G2 arrest and has been associated with therapeutic resistance in human cancers [31], its expression naturally aligns with reduced sensitivity to anti-mitotic agents. The ability of SOLVE to recover this association suggests that it accounts for latent cell-cycle states that obscure such biologically grounded effects in standard models.

Conversely, SOLVE demonstrated greater specificity by filtering out potentially spurious associations that simpler models may flag as significant. For example, while standard logistic regression identified a significant association between NRAS expression and sensitivity to Doxorubicin (*P* < .005), SOLVE found this link to be insignificant (*P* > .1). NRAS activation is a canonical driver of uncontrolled cellular proliferation via the MAPK signaling cascade [32]. Since cytotoxic agents preferentially kill rapidly dividing cells, high proliferation rates can generate broad, non-specific drug sensitivity in large-scale screens, a systematic confounding effect driven by differences in baseline division rate [33]. This correlation reflects a generalized cellular state rather than a specific, mechanistically grounded biomarker of Topoisomerase II inhibition. The association detected by logistic regression therefore likely reflects general pathway activity rather than a direct predictive dependency, whereas SOLVE’s regularized latent-factor framework helps distinguish robust drug-biomarker relationships from confounded ones.

**Figure 4:**
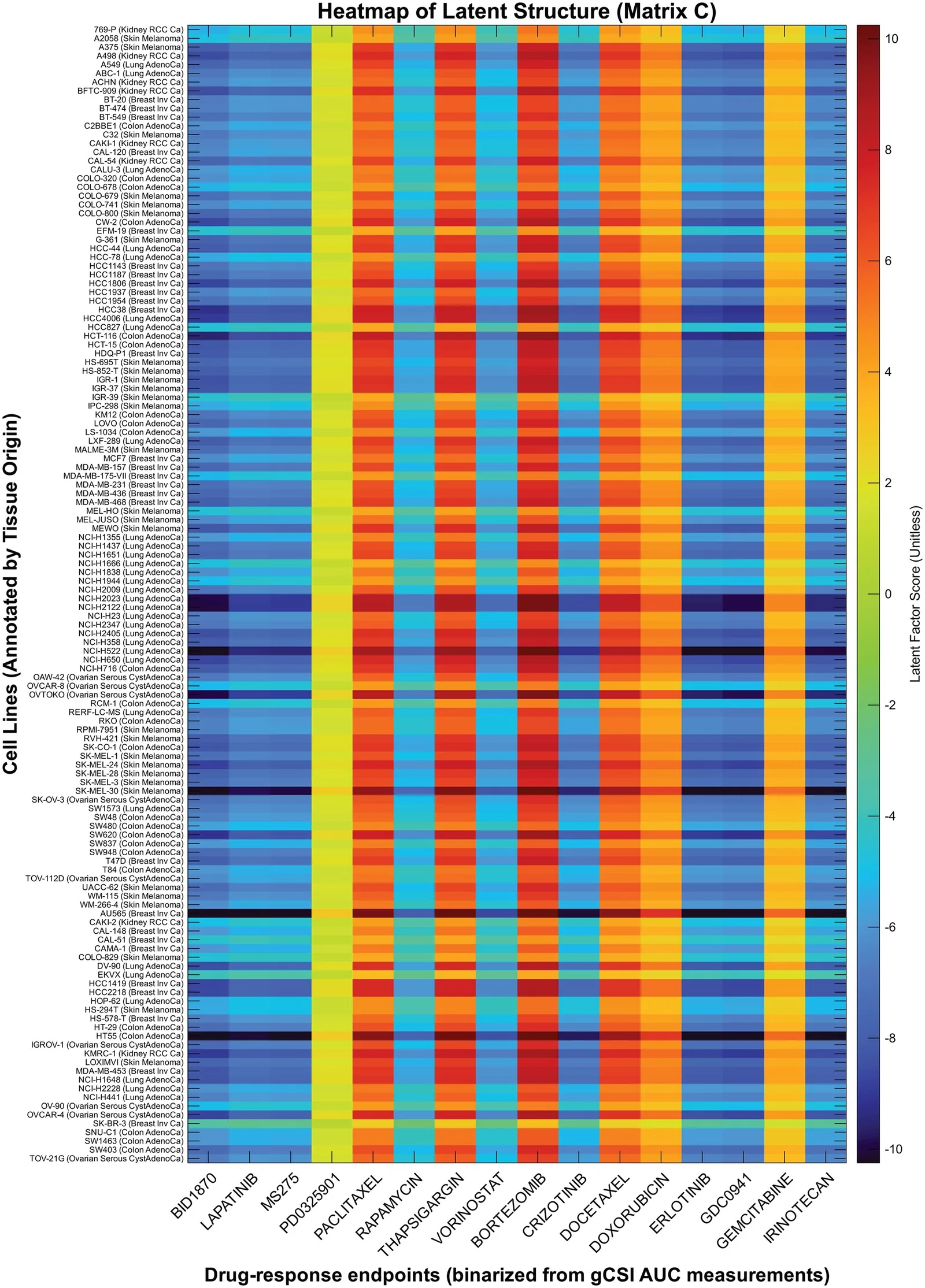
Heatmap of the estimated latent factor matrix of unit rank. Row labels display the names of GCSI cancer cell lines, annotated with their corresponding tissue of origin (disease type). Disease abbreviations: “RCC” (Renal Clear Cell Carcinoma), “AdenoCa” (Adenocarcinoma), “Inv” (Invasive), and “Ca” (Carcinoma). Column labels display the therapeutic compounds corresponding to the drug-response endpoints (binarized from gCSI AUC measurements for classification). The color scale indicates the magnitude of the latent factor score.

Finally, we explored the biological pathway underlying the tuned, rank-1 latent structure found by SOLVE, whose low-rank patterns are visualized in the heatmap in Fig. 4. By design, the latent factor matrix $\hat{C}$ is orthogonal to the model predictors, representing systematic variation in drug response unexplained by the genes retained in *X*. In this experiment, we purposefully excluded explicit intercepts, as the low-rank latent term is capable of absorbing these baseline effects. Inspection shows that no row or column of $\hat{C}$ is constant; hence, the discovered latent factor does not act merely as an intercept, but rather captures meaningful co-variation patterns between groups of cell lines and drugs. The estimated latent matrix was tuned to rank-1, meaning all drug responses are modulated along the same latent direction. Each drug therefore differs only by a single scalar coefficient, which determines the sign and magnitude of its modulation. Consequently, drugs with similar coefficients yield nearly identical column patterns, while drugs with coefficients of opposite sign produce column patterns that are exact opposites.

We performed SVD on the estimate $\hat{C}=udv^{⊤}$, the left singular vector *u* yielding loadings for each cell line. The 10 cell lines with the highest and lowest loadings were selected, and a differential expression analysis was conducted using cBioPortal [34]; the top 100 genes, ranked by *P*-value, were subsequently analyzed for pathway enrichment using Enrichr [35]. This revealed significant enrichment for the Unfolded Protein Response (UPR) pathway across multiple databases, including Reactome Pathways 2024, BioPlanet 2019, and MSigDB Hallmark 2020. This finding is highly relevant, as chronic UPR activation is a known mechanism of drug resistance in cancer [36]. By uncovering this hidden biological state, SOLVE demonstrates its ability to produce more mechanistically interpretable models.

## Conclusion

### Methodological contributions

This work introduces SOLVE, a unified latent-factor framework that jointly models row- and column-specific predictors with a low-rank component for unmeasured variation. In pharmacogenomic analyses, it simultaneously leverages baseline gene expression profiles and drug descriptors while correcting for unobserved confounders—tasks often handled separately by first regressing out measured covariates, then applying PCA to the residuals to capture latent structure.

By enforcing orthogonality between the spaces spanned by observed predictors and the latent structure, the framework ensures identifiability, separating measured effects from residual variation so that latent factors capture only unexplained structure, thus enhancing interpretability.

Computationally, the algorithm alternates truncated SVD updates for the latent term with projection-based coefficient refinements, guided by a quadratic surrogate to efficiently handle general, non-quadratic loss functions. Rank selection uses an elbow method with a degrees-of-freedom penalized criterion to balance fit and complexity, and the framework also supports inference on gene-drug associations via a parametric bootstrap.

As demonstrated in simulations, this integrated approach yields more accurate parameter estimates and more robust rank recovery than competing latent factor models, particularly in high-noise and non-Gaussian settings.

### Pharmacogenomic insights

Applications to large-scale pharmacogenomic datasets (CCLE and GCSI) showed that SOLVE recovers established mechanisms and reveals new, mechanistically plausible associations. SOLVE produced more biologically coherent results by leveraging shared drug descriptors, as demonstrated by its ability to assign nearly identical gene-interaction profiles to two MEK inhibitors with highly correlated molecular features.

Moreover, SOLVE showed increased statistical power, not only recovering established gene-drug associations missed by other latent factor methods—like the links between GRB7 and Erlotinib sensitivity or SMARCD2 and Irinotecan sensitivity—but also uncovering new insights, such as identifying PRR15L as a potential biomarker for downstream pathway dependence on EGFR signaling.

The proposed model also demonstrated greater specificity by correcting spurious associations, such as the finding that NRAS expression was not a significant predictor for Binimetinib sensitivity, in contrast to conventional logistic regression.

Complementing this improved specificity, the model discovered a latent factor in the GCSI data that was significantly enriched for the Unfolded Protein Response (UPR) pathway, a known mechanism of drug resistance.

These findings underscore the value of SOLVE in precision oncology by disentangling measured predictor effects from latent confounding, yielding more robust biomarkers for patient stratification and deeper insight into drug resistance mechanisms.

### Future directions

While motivated by challenges in pharmacogenomics, the SOLVE framework addresses the general problem of matrix outcomes by jointly modeling the effects of both structured predictors (for rows and columns) and unobserved confounders. It is therefore broadly applicable to other settings with this data structure, such as microbial genomics or multi-omics integration.

Promising extensions to the framework include incorporating structured sparsity in the coefficient matrices, for instance, by enforcing row-wise sparsity in matrix *A* for gene selection or column-wise sparsity in matrix *B* for molecular descriptor selection, while preserving the low-rank latent structure. This enhancement would directly address high-dimensional applications by relaxing the assumption that n>p and m>q, enabling simultaneous variable selection and latent structure estimation.

Furthermore, to address the limitation that not all unmeasured confounding is low-rank, another extension could model the residual term as a composite C+S, analogous to Robust PCA [37]. Here, *C* would continue to capture the global, modular confounders, while a sparse matrix *S* would account for local, high-impact but rare events (e.g., a cell-type-specific mutation).

Finally, a further direction is to integrate multi-omics predictors (e.g., mutations, copy number, proteomics) to better disentangle pathway-specific from global stress signals, while leveraging prior biological networks to guide latent factor estimation.

Key Points

- Unified Modeling Framework: SOLVE introduces a unified framework that jointly models row and column predictors with a low-rank latent component in a single, flexible model that handles diverse data types beyond standard regression.
- Guaranteed Identifiability and Interpretability: The framework resolves a critical ambiguity in latent variable modeling by imposing carefully designed orthogonality constraints, forcing the latent component to capture only biological variation unexplained by measured covariates.
- Computationally Efficient and Scalable Algorithm: A computationally efficient algorithm with theoretical guarantees of convergence features closed-form updates, where the latent component is derived via a truncated SVD of a doubly projected residual, yielding a globally optimal solution for regression cases.
- Superior Power for Biomarker Discovery: The method demonstrates superior sensitivity and specificity in pharmacogenomic screens, recovering critical gene-drug associations missed by standard latent factor approaches (such as the link between GRB7/EGFR and EGFR inhibitors) and identifying novel therapeutic candidates.
- Discovery of Latent Biological Mechanisms: The orthogonal latent structure reveals hidden biological drivers of sample response that are not captured by measured features, such as identifying a latent Unfolded Protein Response (UPR) signature as a key drug resistance mechanism in cancer cell lines.

## Data Availability

The pharmacogenomic datasets used in this analysis are openly accessible. The CCLE dataset can be accessed through the Model Organism Development and Evaluation for Cancer (MODAC) portal under asset ID NCI-DME-MS01-8088592 with the path/NCI_DOE_Archive/JDACS4C/JDACS4C_Pilot_1/cancer_drug_response_prediction_dataset/top_6.res_reg.cf_rnaseq.dd_dragon7.labled.csv.gz. The GCSI dataset is available through the same MODAC portal. The underlying code for SOLVE, including algorithms for rank tuning, model fitting, and bootstrap inference, is available on Code Ocean at https://doi.org/10.24433/CO.3000218.v1 [38].

## Appendix A

**Proof of Theorem 1** 

*Proof.* The loss can be decomposed into orthogonal components, which allows us to isolate the term involving $C_{0}$ and solve it independently. By inserting the identity matrices $I=P_{X}+P_{X}^{⊥}$ and $I=P_{Z^{⊤}}+P_{Z^{⊤}}^{⊥}$,

$$\begin{array}{cc} & Y-XA_{0}-P_{X}^{⊥}B_{0}Z\\ & =(P_{X}+P_{X}^{⊥})(Y-XA_{0}-P_{X}^{⊥}B_{0}Z)(P_{Z^{⊤}}+P_{Z^{⊤}}^{⊥})\\ & =P_{X}(Y-XA_{0}-P_{X}^{⊥}B_{0}Z)P_{Z^{⊤}}+P_{X}(Y-XA_{0}-P_{X}^{⊥}B_{0}Z)P_{Z^{⊤}}^{⊥}\\ & \;+P_{X}^{⊥}(Y-XA_{0}-P_{X}^{⊥}B_{0}Z)P_{Z^{⊤}}+P_{X}^{⊥}(Y-XA_{0}-P_{X}^{⊥}B_{0}Z)P_{Z^{⊤}}^{⊥}. \end{array}$$

The Pythagorean theorem for the Frobenius norm yields

$$\begin{array}{c} l(\eta )=\frac{1}{2}∥P_{X}^{⊥}(Y-XA_{0}-P_{X}^{⊥}B_{0}Z)P_{Z^{⊤}}^{⊥}-P_{X}^{⊥}C_{0}P_{Z^{⊤}}^{⊥}{∥}_{F}^{2}\\ +\frac{1}{2}∥P_{X}(Y-XA_{0}-P_{X}^{⊥}B_{0}Z)P_{Z^{⊤}}{∥}_{F}^{2}\\ +\frac{1}{2}∥P_{X}(Y-XA_{0}-P_{X}^{⊥}B_{0}Z)P_{Z^{⊤}}^{⊥}{∥}_{F}^{2}\\ +\frac{1}{2}∥P_{X}^{⊥}(Y-XA_{0}-P_{X}^{⊥}B_{0}Z)P_{Z^{⊤}}{∥}_{F}^{2}\\ =\frac{1}{2}∥P_{X}^{⊥}YP_{Z^{⊤}}^{⊥}-P_{X}^{⊥}C_{0}P_{Z^{⊤}}^{⊥}{∥}_{F}^{2}+c(A_{0},B_{0}), \end{array}$$

where $c(A_{0},B_{0})$ are independent of $C_{0}$. To determine the optimal ${\hat{C}}_{0}$, we only need to solve

(18)
$$\underset{C_{0}}{\min}\frac{1}{2}∥P_{X}^{⊥}YP_{Z^{⊤}}^{⊥}-P_{X}^{⊥}C_{0}P_{Z^{⊤}}^{⊥}{∥}_{F}^{2}\;s.t.\;\text{rank}(C_{0})\leq r$$

Let $\tilde{Y}=P_{X}^{⊥}YP_{Z^{⊤}}^{⊥}$ and $l_{0}(C_{0})=∥\tilde{Y}-P_{X}^{⊥}C_{0}P_{Z^{⊤}}^{⊥}{∥}_{F}^{2}$/2. By the Eckart–Young–Mirsky theorem [39],

$$l_{0}(C_{0})\geq l_{0}(S(\tilde{Y};r)),\forall C_{0}:\text{rank}(C_{0})\leq r.$$

Let $\tilde{C}=S(\tilde{Y};r)$. We claim that

(19)
$$P_{X}^{⊥}{\tilde{C}}_{0}={\tilde{C}}_{0},\;{\tilde{C}}_{0}P_{Z^{⊤}}^{⊥}={\tilde{C}}_{0},$$

or ${\tilde{C}}_{0}=P_{X}^{⊥}{\tilde{C}}_{0}P_{Z^{⊤}}^{⊥}$, and thus ${\tilde{C}}_{0}$ is a global minimizer of 18. Indeed, letting the SVD of $\tilde{Y}$ be $\tilde{Y}=UDV^{⊤}$, it is easy to verify that

$${\tilde{C}}_{0}=S(\tilde{Y};r)=U_{r}D_{r}V_{r}^{⊤}=UDV^{⊤}V_{r}V_{r}^{⊤}=\tilde{Y}V_{r}V_{r}^{⊤}.$$

Together with $P_{X}^{⊥}\tilde{Y}=\tilde{Y}$, we have $P_{X}^{⊥}{\tilde{C}}_{0}={\tilde{C}}_{0}$. A similar argument shows $S(\tilde{Y};r)=U_{r}U_{r}^{⊤}UDV^{⊤}=U_{r}U_{r}^{⊤}\tilde{Y}$ or ${\tilde{C}}_{0}P_{Z^{⊤}}^{⊥}={\tilde{C}}_{0}$.

Next, we solve for the optimal $\left({\hat{A}}_{0},{\hat{B}}_{0}\right)$. Let $\bar{Y}=Y-{\hat{C}}_{0}$. Owing to the convexity and differentiability of loss

$$f(A_{0},B_{0};\bar{Y}):=\frac{1}{2}∥\bar{Y}-XA_{0}-P_{X}^{⊥}B_{0}Z{∥}_{F}^{2},$$

the optimal solutions are characterized by $\nabla_{A_{0}}f(A_{0},B_{0})=0,\nabla_{B_{0}}f(A_{0},B_{0})=0$. It is straightforward to obtain $\nabla_{A_{0}}f(A_{0},B_{0})=X^{⊤}(XA_{0}-(\bar{Y}-P_{X}^{⊥}B_{0}Z))$. For $\nabla_{B_{0}}f$, we use the expansion:

$$\begin{array}{c} f(A_{0},B_{0}+\Delta ;\bar{Y})=\frac{1}{2}∥P_{X}^{⊥}(B_{0}+\Delta )Z-(\bar{Y}-XA_{0}){∥}_{F}^{2}\\ =f(A_{0},B_{0};\bar{Y})+\langle P_{X}^{⊥}\Delta Z,P_{X}^{⊥}B_{0}Z-(\bar{Y}-XA_{0})\rangle +O(∥\Delta {∥}_{F}^{2})\\ =f(A_{0},B_{0};\bar{Y})+\langle \Delta ,P_{X}^{⊥}(P_{X}^{⊥}B_{0}Z-(\bar{Y}-XA_{0}))Z^{⊤}\rangle +O(∥\Delta {∥}_{F}^{2})\\ =f(A_{0},B_{0};\bar{Y})+\langle \Delta ,P_{X}^{⊥}B_{0}ZZ^{⊤}-P_{X}^{⊥}\bar{Y}Z^{⊤}\rangle +O(∥\Delta {∥}_{F}^{2}) \end{array}$$

yielding $\nabla_{A_{0}}f(A_{0},B_{0})=P_{X}^{⊥}B_{0}ZZ^{⊤}-P_{X}^{⊥}\bar{Y}Z^{⊤}$. Therefore,

(20)
$$X^{⊤}X{\hat{A}}_{0}=X^{⊤}\bar{Y}$$

(21)
$$P_{X}^{⊥}{\hat{B}}_{0}ZZ^{⊤}=P_{X}^{⊥}\bar{Y}Z^{⊤}.$$

Together with 19, the conclusion of the theorem follows. □

**Proof of Theorem 2** 

*Proof.* By definition,

$$g(\eta^{\left(t+1\right)},\eta^{\left(t\right)})\leq g(\eta^{\left(t\right)},\eta^{\left(t\right)})=l(\eta^{\left(t\right)}).$$

Moreover, by the fundamental theorem of calculus, we have

$$\begin{array}{c} g(\eta^{\left(t+1\right)},\eta^{\left(t\right)})-l(\eta^{\left(t+1\right)})\\ =l(\eta^{\left(t\right)})+\langle \nabla l(\eta^{\left(t\right)}),\eta^{\left(t+1\right)}-\eta^{\left(t\right)}\rangle -l(\eta^{\left(t+1\right)})+\frac{\rho }{2}∥\eta^{\left(t+1\right)}-\eta^{\left(t\right)}{∥}_{F}^{2}\\ =\frac{\rho }{2}∥\eta^{\left(t+1\right)}-\eta^{\left(t\right)}{∥}_{F}^{2}-(l(\eta^{\left(t+1\right)})-l(\eta^{\left(t\right)})-\langle \nabla l(\eta^{\left(t\right)}),\eta^{\left(t+1\right)}-\eta^{\left(t\right)}\rangle )\\ =\frac{\rho }{2}∥\eta^{\left(t+1\right)}-\eta^{\left(t\right)}{∥}_{F}^{2}-(l(\eta^{\left(t\right)}+s(\eta^{\left(t+1\right)}-\eta^{\left(t\right)})){|}_{0}^{1}-\int_{0}^{1}\langle \nabla l(\eta^{\left(t\right)}),\eta^{\left(t+1\right)}-\eta^{\left(t\right)}\rangle )ds)\\ =\frac{\rho }{2}∥\eta^{\left(t+1\right)}-\eta^{\left(t\right)}{∥}_{F}^{2}-\int_{0}^{1}\frac{\partial }{\partial s}l(\eta^{\left(t\right)}+s(\eta^{\left(t+1\right)}-\eta^{\left(t\right)}))-\langle \nabla l(\eta^{\left(t\right)}),\eta^{\left(t+1\right)}-\eta^{\left(t\right)}\rangle )ds\\ =\frac{\rho }{2}∥\eta^{\left(t+1\right)}-\eta^{\left(t\right)}{∥}_{F}^{2}-\int_{0}^{1}\langle \nabla l(\eta^{\left(t\right)}+s(\eta^{\left(t+1\right)}-\eta^{\left(t\right)}))-\nabla l(\eta^{\left(t\right)}),\eta^{\left(t+1\right)}-\eta^{\left(t\right)}\rangle )ds\\ \geq \frac{\rho }{2}∥\eta^{\left(t+1\right)}-\eta^{\left(t\right)}{∥}_{F}^{2}-\int_{0}^{1}∥\nabla l(\eta^{\left(t\right)}+s(\eta^{\left(t+1\right)}-\eta^{\left(t\right)}))-\nabla l(\eta^{\left(t\right)}){∥}_{F}∥\eta^{\left(t+1\right)}-\eta^{\left(t\right)}\rangle {∥}_{F}ds\\ \geq \frac{\rho }{2}∥\eta^{\left(t+1\right)}-\eta^{\left(t\right)}{∥}_{F}^{2}-\int_{0}^{1}Ls∥\eta^{\left(t+1\right)}-\eta^{\left(t\right)}{∥}_{F}∥\eta^{\left(t+1\right)}-\eta^{\left(t\right)}\rangle {∥}_{F}ds\\ =\frac{\rho -L}{2}∥\eta^{\left(t+1\right)}-\eta^{\left(t\right)}{∥}_{F}^{2}, \end{array}$$

where the first inequality follows from the Cauchy–Schwarz inequality and the second is a consequence of the Lipschitz gradient condition. The conclusion thus follows. □
